# Iterating a framework for the prevention of caregiver depression in dementia: a multi-method approach

**DOI:** 10.1017/S1041610217002629

**Published:** 2017-12-10

**Authors:** Jiangbo Ying, Philip Yap, Mihir Gandhi, Tau Ming Liew

**Affiliations:** 1 Department of Geriatric Psychiatry, Institute of Mental Health, Singapore; 2 Department of Geriatric Medicine, Khoo Teck Puat Hospital, Singapore; 3 Geriatric Education and Research Institute, Singapore; 4 Biostatistics, Singapore Clinical Research Institute, Singapore; 5 Centre for Quantitative Medicine, Duke-NUS Medical School, Singapore; 6 Psychotherapy unit, Institute of Mental Health, Singapore; 7 Saw Swee Hock School of Public Health, National University of Singapore, Singapore

**Keywords:** dementia, caregiver, depression, prevention, framework

## Abstract

**Background:**

Dementia caregiving is often stressful and depression in family caregivers is not uncommon. As caregiver depression can have significant effects, there is a need for preventive efforts which are consistent with the extensive literature. We sought to consolidate the wide range of evidence (using a multi-method approach) into a simple framework that can guide the prevention of caregiver depression.

**Methods:**

Using multiple logistic regression, we derived the predictors of caregiver depression from an empirical dataset containing key information and depression scores (based on the Center-for-Epidemiological-Studies-Depression-Scale) of 394 family caregivers. We then chose an underpinning theory as the foundation of the framework, and conducted an umbrella systematic review to find possible links between the derived predictors and the theory. Last, we compared the iterated framework with known interventions for caregiver depression in recent literature to assess whether the framework could map meaningfully with the known interventions.

**Results:**

Significant predictors of caregiver depression included primary caregiver (odds ratio, OR = 1.53), severe dementia (OR = 1.40), and behavioral problems (OR = 3.23), lower education (OR = 1.77), and spousal caregivers (OR = 1.98). The integrated framework derived focuses on four strategic areas: physical-care demands of persons with dementia (PWD), behavioral problems of PWD, caregiving competency, and loss and grief of caregivers. This framework is supported by known interventions for caregiver depression in recent literature.

**Conclusions:**

By consolidating a broad range of evidence, we iterated a framework to aid the understanding and prevention of caregiver depression in dementia. The framework offers an approach to prevention which is simple, systematic, and reflective of the extensive literature.

## Introduction

Depression occurs in at least one in three caregivers of persons with dementia (PWD), as reported in a recent meta-analysis (Sallim *et al.*, 2015). This is comparatively higher than those found in the general population, or in the caregivers of other physical or mental illnesses (Sallim *et al.*, 2015). Depression can cause a variety of psychological and somatic problems to the caregivers, and increase the risk of caregivers contemplating suicide (O'Dwyer *et al.*, 2016). It compromises caregivers’ physical health (Pinquart and Sorensen, 2007) and has been shown to cause the caregivers to place PWD in an institutional care facility more rapidly (Coehlo *et al.*, 2007). Depression in caregivers can also impact the PWD adversely, as it has been associated with more rapid cognitive decline (Norton *et al.*, 2013) and depression in PWD (Teri *et al.*, 1997).

Considering the significant impact of caregiver depression, it is pertinent to address this issue not just by providing interventions after the depression has manifested, but by increasing pro-active efforts to prevent caregiver depression before it can cause any adverse effects. To be effective, such prevention efforts should ideally be grounded in an evidence-based framework which is consistent with the extensive literature, including those relating to the predictors, the underpinning theory, and the interventions of caregiver depression. However, no such framework exists to date. While much has been known about caregiver depression, there has been little effort to consolidate the extensive literature into a simple and user-friendly structure that can be useful to busy practitioners and policy-makers. Individuals have largely been left to themselves to plow through the wide range of evidence for relevant information on the prevention of caregiver depression in dementia.

In this study, we sought to integrate the broad range of evidence into a simple framework that can guide the prevention efforts of caregiver depression. To incorporate the evidence on the various aspects of caregiver depression, we chose a multi-method approach which included (1) deriving the predictors of caregiver depression using an empirical dataset; (2) utilizing an underpinning theory to help us understand caregiver depression and build the foundation of the framework; (3) conducting an umbrella systematic review (that is, a systematic review of review articles) to find the links between the derived predictors and the underpinning theory; and (4) comparing the iterated framework with known interventions for caregiver depression in recent literature to assess whether the framework could map meaningfully with the known interventions.

## Methods

### Derivation of predictors of caregiver depression using an empirical dataset

To derive the predictors of caregiver depression, we used an empirical dataset containing key information related to 394 spousal or children caregivers of community-dwelling PWD from the dementia services of two tertiary hospitals which serve the population in the North-East of Singapore. The information was collected through a cross-sectional study using consecutive sampling method, with a response rate of 87.8% in the recruitment. This study has previously received ethical approval from the Domain Specific Review Board of Singapore.

In this dataset, the Center for Epidemiological Studies Depression Scale (CES-D) was used to measure caregiver depression in dementia. It is a 20-item, self-administered scale which measures depressive symptomatology in the previous week (Radloff, 1977). Each item is scored on a four-point Likert scale to reflect the frequency of each depressive symptom and the total score ranges from 0 to 60. In a recent meta-analysis, CES-D has been shown to have good utility for the diagnosis of major depression – it has an area under the curve of 0.87 on the summary receiver operating characteristic curve, with sensitivity of 0.87 and specificity of 0.70 at the recommended cut-off score of 16 (Vilagut *et al.*, 2016).

The dataset also captured information related to the caregiver and PWD. Those relating to the caregiver included age, gender, ethnicity, marital status, employment status, education, relationship with PWD, whether the caregiver is staying with the PWD, duration of caregiving, frequency of caregiving, and role as primary caregiver. Those relating to the PWD included age, gender, duration of dementia diagnosis, age of the PWD when dementia was first diagnosed, stage of dementia, and presence of severe behavioral problem. The stage of dementia was captured using a brief measure based on the descriptions of the three dementia severities described in the revised third edition of Diagnostic and Statistical Manual of Mental Disorders (DSM-III-R) (American Psychiatric Association, 1987). From the three options, participants chose the description that best described the PWD – still capable of independent living (mild stage), needs some assistance with daily living (moderate stage), or needs round-the-clock supervision (severe stage). This brief measure was previously shown to have reasonable agreement with Clinical Dementia Rating Scale (kappa 0.56–0.6) (Forsell *et al.*, 1992; Juva *et al.*, 1994), which is one of the most commonly used scale to stage dementia (Morris, 1993; Rikkert *et al.*, 2011). This brief measure is also nearly identical to the re-introduced dementia severity in DSM-5 (American Psychiatric Association, 2013). The presence of severe behavioral problem was indirectly measured through the need for admission to the geriatric psychiatry ward, indicating a behavioral problem that was too severe to be managed in the community setting.

To investigate the predictors of caregiver depression, we first performed simple logistic regression to identify factors associated with significant depression in caregivers (CES-D score ≥16). All variables with *p* ≤ 0.20 in the simple regression were then entered into multiple regression and variables with *p* > 0.15 in multiple regression were removed through backward variable selection method (Grobbee and Hoes, 2014). In the final model, variables with *p* ≤ 0.05 were considered as significant predictors, while variables with *p* values between 0.05 and 0.15 were included as probable predictors. Less stringent cut-off for *p* values was chosen to allow the development of a framework which is more inclusive to encompass predictors with even a small influence on caregiver depression. The goodness of fit of the final regression model was assessed with the Hosmer–Lemeshow test. The statistical analyses were performed using STATA software version 13.

### Iteration of a framework for the prevention of caregiver depression

To develop a prevention framework, we first used the transactional model of stress and coping (Lazarus and Folkman, 1984) as the foundation upon which we built our understanding of caregiver depression. This model has been commonly used to understand the experience of burden and depression in caregivers of PWD (van der Lee *et al.*, 2014). It posits that stress appraisal and coping are the two key processes and mediators of the ongoing relationship between an external event and the person (Folkman, 2013). An event is considered stressful when it is perceived as personally significant, and when it exceeds the person's ability to manage the situation or to manage the distress that arises from the situation.

We sought to understand how the derived predictors from this study relate to the transactional model of stress and coping. For this purpose, we reviewed the literature using the methodology of umbrella systematic review (that is, systematic review of review articles) to find possible factors that may mediate the relationship between the predictors and those of stress and coping. We searched PubMed, Embase, and PsycINFO for systematic reviews on observational or qualitative studies with keywords pertaining to “dementia caregiving,”, “coping,” and those related to the derived predictors from this study. The search strategies are shown in (Supplementary Appendix A1, available as supplementary material attached to the electronic version of this paper at www.journals.cambridge.org/jid_IPG). In the study selection, we excluded studies which were not systematic reviews, studies which were not focused on dementia caregiving, and studies which focused on interventions. From the selected studies, we extracted all data which were related to our predictors of interest. The study selection and data extraction were conducted independently by two of our researchers, with disagreements resolved through consensus. With the extracted data from the umbrella systematic review, we iterated a framework that can integrate the predictors with the transactional model of stress and coping through plausible mediating factors.

We then evaluated the coherence of the proposed framework by comparing it to known interventions for caregiver depression in recent literature and assessing whether the framework can produce a meaningful mapping of the interventions. For this purpose, we utilized the search results of a recent systematic review (Weinbrecht *et al.*, 2016) on caregiver depression in dementia which identified 33 randomized controlled trials (RCTs) in the last decade and demonstrated a modest yet significant benefit of interventions in alleviating the depressive symptoms of caregivers (standardized mean difference 0.13, 95% CI 0.03–0.23). Because most of the RCTs involved multi-component interventions, two of our researchers independently reviewed all the 33 RCTs to break down the multi-component interventions into individual components of interventions, before we compare the interventions with our proposed framework.

## Results

Table 1 shows the demographic information of the caregivers from our empirical dataset and the odds ratios (OR) of predictors of caregiver depression from simple logistic regression. In multiple regression, predictors of caregiver depression included primary caregiver (OR 1.53, 95% CI 0.96–2.45, *p* = 0.074), caring for PWD with later stage of disease (OR 1.40, 95% CI 0.92–2.13, *p* = 0.115), caring for PWD with severe behavioral problems (OR 3.23 95% CI 1.20–8.73, *p* = 0.020), secondary or below education (OR 1.77, 95% CI 1.12–2.81, *p* = 0.015), and spousal relationship (OR 1.98, 95% CI 1.06–3.71, *p* = 0.033). This final model showed a good fit in the Hosmer–Lemeshow test (*p* = 0.752).

**Table 1.** Demographic information of the caregivers and the persons with dementia, and the association with caregiver depression in simple logistic regression (*n* = 394)

variable	*n* (%)	or (95% ci)^a^	*p* value^a^
VARIABLES RELATED TO CAREGIVERS			
Age, mean (SD)	53.0 (10.7)	1.02 (1.00–1.03)	**0.105**
Female gender	236 (59.9)	1.22 (0.81–1.83)	0.344
Ethnic			0.291
Chinese	341 (86.6)	Ref.	
Malay	25 (6.3)	2.00 (0.87–4.59)	
Indian	18 (4.6)	1.67 (0.64–4.33)	
Others	10 (2.5)	1.34 (0.38–4.70)
Marital status			0.420
Married	271 (68.8)	Ref.	
Single	94 (23.9)	0.85 (0.53–1.37)	
Widowed/divorced/separated	29 (7.3)	0.61 (0.27–1.35)	
Employment status			**0.031**
Not working	123 (31.2)	Ref.	
Working part-time	52 (13.2)	0.89 (0.47–1.71)	
Working full-time	219 (55.6)	0.57 (0.36–0.88)	
Highest education			**0.005**
Primary or no formal education	41 (10.4)	Ref.	
Secondary	228 (57.9)	0.85 (0.44–1.65)	
Tertiary	125 (31.7)	0.42 (0.21–0.86)	
Relationship with PWD			**0.002**
Child	340 (86.3)	Ref.	
Spouse	54 (13.7)	2.60 (1.43–4.73)	
Staying with PWD	264 (67.0)	1.33 (0.87–2.03)	**0.191**
Duration of caregiving in years, mean (SD)	6.8 (6.7)	0.99 (0.96–1.02)	0.373
Frequency of caregiving			0.231
Daily, for at least 4 hours a day	211 (53.6)	Ref.	
Daily, but less than 4 hours a day	79 (20.0)	0.74 (0.44–1.24)	
At least once a week	84 (21.3)	0.60 (0.36–1.01)	
Less than once a week	20 (5.1)	0.69 (0.27–1.74)	
Primary caregiver role	279 (70.8)	1.69 (1.08–2.65)	**0.021**
VARIABLES RELATED TO PWD			
Age, mean (SD)	79.5 (8.2)	0.98 (0.95–1.00)	**0.079**
Female gender	278 (70.6)	0.85 (0.55–1.32)	0.479
Age at dementia diagnosis, mean (SD)	75.6 (8.5)	0.99 (0.96–1.01)	0.215
Duration of dementia diagnosis in years, mean (SD)	4.5 (3.5)	0.97 (0.92–1.03)	0.278
Stage of dementia ^b^			**0.087**
Mild	62 (15.7)	Ref.	
Moderate	163 (41.4)	1.18 (0.65–2.16)	
Severe	169(42.9)	1.76 (0.97–3.19)	
Severe behavioral problem ^c^	22 (5.6)	3.53 (1.35–9.23)	**0.010**

OR, odds ratio; 95% CI, 95% confidence interval; SD, standard deviation; PWD, persons with dementia; ref., reference group in logistic regression.

a Derived from simple logistic regression with CES-D≥16 as the dependent variable. Bold-faced *p* values are ≤0.20.

b We obtained a brief measure of the stage of dementia using the three dementia severities described in the revised third edition of Diagnostic and Statistical Manual of Mental Disorders (DSM-III-R). From the three options, participants chose the description that best described the PWD – still capable of independent living (mild stage), needs some assistance with daily living (moderate stage), or needs round-the-clock supervision (severe stage).

c The presence of severe behavioral problem was indirectly measured through the need for admission to a geriatric psychiatry ward, indicating a behavioral problem that was too severe to be managed in the community setting.

**Figure 1.**
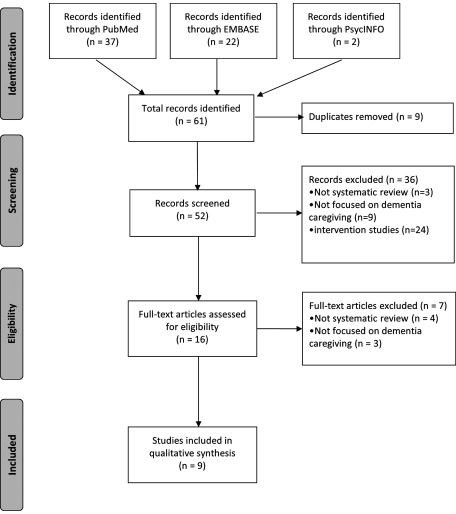
Flowchart of selection process in our umbrella systematic review.

**Figure 2.**
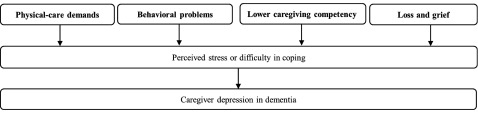
A proposed framework to facilitate the prevention efforts of caregiver depression. The key factors of caregiver depression are bold faced in the figure.

In the umbrella systematic review, we identified nine review articles which are related to our derived predictors and the transactional model of stress and coping. The flowchart of the selection process is shown in Figure 1. The characteristics and key findings of these review articles are summarized in Table 2. Using the key findings from the review articles (Table 2), we attempted to find the links between our derived predictors of caregiver depression and the transactional model of stress and coping (Table 3). We then iterated a framework that allows integration between our derived predictors and the transactional model of stress and coping. The framework is illustrated in Figure 2, while the iterative and inferential processes of the framework are further described in the paragraph below.

**Table 2.** Key findings of the nine review articles identified from our umbrella systematic review

review article (first author and year)	number of studies included in the review (type of studies)	aims of the review article	key findings related to our predictors of interest
Cabote *et al*. (2015)	5 (qualitative)	Describe the experiences of family caregivers of caring for a person with younger onset dementia	1. Spousal caregivers notice the changes in the person with dementia long before the actual diagnosis, and it creates a sense of uncertainty in the spousal caregivers. 2. Spousal caregivers experience grief and loss in the context of the present as well as in the future. 3. Spousal caregivers experience a diminished reciprocity in the relationship with the person with dementia, and they also needed to renegotiate the sexual roles in the marriage. 4. Spousal caregivers experience burden in caring for the person with dementia. 5. Spousal caregivers have difficulty letting go of the person with dementia to allow others to gradually take over the care.
Caceres *et al*. (2016)	8 (quantitative) 3 (qualitative)	1. Evaluate the experience of family caregivers of persons with frontotemporal dementia. 2. Explore the impact of caregiving on family caregivers’ health and well-being.	1. Frequency of behavioral disturbances was the primary predictor of negative emotions and caregiver burden. 2. Spousal caregivers had significantly greater rates of depression. 3. Spousal caregivers experience feelings of isolation and loss of self-esteem due to the emotional distance with the person with dementia.
Chiao *et al*. (2015)	21 (quantitative)	Identify the main factors of caregiver burden among the informal caregivers of people with dementia living in the community.	1. Behavioral disturbances in patients with dementia were associated with greater burden in family caregivers. 2. Worsening severity of dementia and poor functional status were associated with primary caregiver experiencing greater burden. 3. Caregivers with low educational level were associated with greater burden. 4. Caregivers who had a relatively heavy patient care load experienced a greater burden from their caregiving. 5. Spouse caregivers experienced greater burden.
Connell and Gibson (1997)	12 (quantitative)	Examine the effect of race, culture, and ethnicity on the dementia caregiving experience.	1. Impairment in physical activities of daily living predicted burden in Black caregivers, while impairment in instrumental activities of daily living predicted burden in White caregivers. 2. Spouse caregivers reported highest levels of stress, followed by adult children and other family caregivers.
Gilhooly *et al*. (2016)	45 (systematic reviews)	Summarize all systematic reviews related to stress and coping in dementia caregiving.	1. Pooled correlations indicated moderate associations between BPSD (behavioral and psychological symptoms of dementia) and caregiver burden, caregiver distress, and caregiver depression. 2. A wide range of symptoms in persons with dementia was associated with caregiver depression and burden.
			3. Patients’ behavioral problems and caregivers’ competence were among the most consistent determinants of caregiver burden, depression, and mental health. Behavioral problems were more significant than cognitive disorders or lack of self-care. Caregivers’ feeling of competence or higher self-efficacy was beneficial with regards to burden and mental health.
Pozzebon *et al*. (2016)	16 (qualitative)	Synthesize the results of qualitative studies that have explored the lived experience of spousal caregivers of persons with dementia.	1. The theme of “loss of partner” was central to spousal caregivers, and around this central experience spouses described various processes: acknowledging change, being in crisis, adapting and adjusting, accepting, and moving forward.
Roche *et al*. (2016)	21 (quantitative)	Investigate the caregiver and care-recipient factors that predict the adaptive and maladaptive use of coping strategies by spousal caregivers of persons with dementia.	1. Higher caregiver education predicted solution-focused coping, while lower caregiver education predicted emotional support/acceptance-based coping. 2. Behavioral problems in persons with dementia predicted dysfunctional coping in caregivers. 3. Caregivers of persons with greater independence in ADLs used more solution-focused coping, whereas caregivers of persons with less independence in ADLs employed emotional support/acceptance-based coping.
Van der Lee *et al*. (2014)	32 (quantitative)	Evaluate patient and caregiver characteristics that determine subjective caregiver burden or caregiver depression.	1. Behavioral problems increased burden and depressive symptoms in caregivers. 2. Disabilities in activities of daily living increased burden and depressive symptoms in caregivers. 3. Lower caregiving competence increased burden and depressive symptoms in caregivers.
Wadham *et al*. (2016)	10 (qualitative)	Synthesize qualitative studies exploring the relationship between dementia and couple relationship.	1. Dementia changes how couples connect with each other, with some spouses gradually feeling the loss of closeness and shared identity. 2. Changes in cognitive ability of the person with dementia impacted upon their ability to perform daily tasks, thus requiring caregivers to take on more responsibility to compensate. 3. Different couples cope with dementia in different ways. Some of the coping strategies are more adaptive while some are less so.

**Table 3.** The link between our predictors of caregiver depression and the transactional model of stress and coping

our predictors of interest	relevant findings from our umbrella systematic review	our conclusion on the link
Primary-caregiving role and caring for PWD at later stage of disease	• Caregivers who had a relatively heavy patient care load experienced a greater burden from their caregiving (Chiao *et al*., 2015). • Impairment in physical activities of daily living predicted burden in Black caregivers, while impairment in instrumental activities of daily living predicted burden in White caregivers (Connell and Gibson, 1997). • Caregivers of persons with greater independence in ADLs used more solution-focused coping, whereas caregivers of persons with less independence in ADLs employed emotional support/acceptance-based coping (Roche *et al*., 2016). • Disabilities in activities of daily living increased burden and depressive symptoms in caregivers (van der Lee *et al*., 2014).	Physical-care demands
Caring for PWD with behavioral problems	• Frequency of behavioral disturbances was the primary predictor of negative emotions and caregiver burden (Caceres *et al*., 2016). • Behavioral disturbances in patients with dementia were associated with greater burden in family caregivers (Chiao *et al*., 2015). • Pooled correlations indicated moderate associations between BPSD (behavioral and psychological symptoms of dementia) and caregiver burden, caregiver distress, and caregiver depression (Gilhooly *et al*., 2016). • Behavioral problems in persons with dementia predicted dysfunctional coping in caregivers (Roche *et al*., 2016). • Behavioral problems increased burden and depressive symptoms in caregivers (van der Lee *et al*., 2014).	Behavioral problems
Lower education	• Caregivers with low educational level were associated with greater burden (Chiao *et al*., 2015). • Higher caregiver education predicted solution-focused coping, while lower caregiver education predicted emotional support/acceptance-based coping (Roche *et al*., 2016). • Caregivers’ competence was among the most consistent determinant of caregiver burden, depression, and mental health. Caregivers’ feeling of competence or higher self-efficacy was beneficial with regards to burden and mental health (Gilhooly *et al*., 2016). • Lower caregiving competence increased burden and depressive symptoms in caregivers (van der Lee *et al*., 2014).	Lower caregiving competency
Spousal relationship	• Spousal caregivers experience grief and loss in the context of the present as well as in the future (Cabote *et al*., 2015). • Spousal caregivers experience feelings of isolation and loss of self-esteem due to the emotional distance with the person with dementia (Caceres *et al*., 2016). • The theme of “loss of partner” was central to spousal caregivers, and around this central experience spouses described various processes: acknowledging change, being in crisis, adapting and adjusting, accepting, and moving forward (Pozzebon *et al*., 2016). • Dementia changes how couples connect with each other, with some spouses gradually feeling the loss of closeness and shared identity (Wadham *et al*., 2016).	Loss and grief

PWD, persons with dementia.

Physical-care demands on the caregiver, reported in four of the review articles (Connell and Gibson, 1997; van der Lee *et al.*, 2014; Chiao *et al.*, 2015; Roche *et al.*, 2016) as high care load or caring for PWD with impairments in activities of daily living, were shown to affect the perceived stress and coping of caregivers. In the development of caregiver depression, we postulate that physical-care demands may mediate the effect of primary-caregiving role and severe dementia (the predictors from our study), since primary caregivers are generally faced with a host of care responsibilities while persons with severe dementia require more assistance from caregivers in their activities of daily living. Behavioral problems in PWD, a predictor in this study, has also been reported to affect the perceived stress and coping of caregivers in five of the review articles (van der Lee *et al.*, 2014; Chiao *et al.*, 2015; Caceres *et al.*, 2016; Gilhooly *et al.*, 2016; Roche *et al.*, 2016). While two review articles reported the effect of educational attainment on stress and coping (Chiao *et al.*, 2015; Roche *et al.*, 2016), it was less clear what mediated this effect. We can only postulate that caregiving competency may possibly mediate the effect of lower education (the predictor from our study) on caregiver depression. Caregiving competency has been reported to affect the perceived stress of caregivers in two of the review articles (van der Lee *et al.*, 2014; Gilhooly *et al.*, 2016). While there has not been a direct link in the literature, it can be possible that some caregivers with less education may have difficulty in mastering the more complex skills required to care for the PWD. Four reviews articles (Cabote *et al.*, 2015; Caceres *et al.*, 2016; Pozzebon *et al.*, 2016; Wadham *et al.*, 2016) alluded to the experience of loss and grief of spousal caregivers, with one of them highlighting loss and grief as the central theme of the spouses’ lived experience (Pozzebon *et al.*, 2016). We postulate that the experience of loss and grief may be the mediating factor between spousal caregivers (the predictor of our study) and depression, as the continual contention with the difficult experience of loss and grief may leave caregivers with little resources to cope with the stress of caregiving.

**Figure 3.**
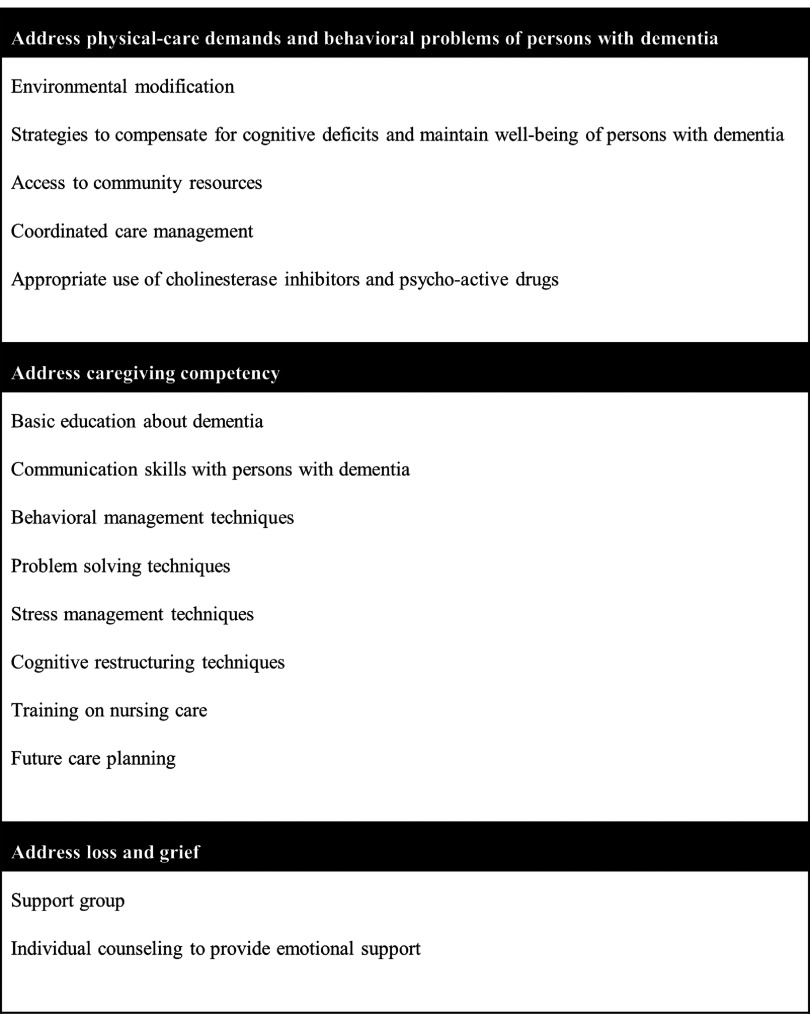
Known interventions for caregiver depression in the literature, classified according to the key factors from our proposed framework.

We evaluated the coherence of the proposed framework by comparing it to known interventions for caregiver depression in recent literature. Using the 33 RCTs from a recent systematic review (Weinbrecht *et al.*, 2016) as reference, we identified 15 unique components of interventions for caregiver depression that have been used in the last decade. The breakdown of the individual components of interventions is shown in (Supplementary Appendix A2, available as supplementary material attached to the electronic version of this paper at www.journals.cambridge.org/jid_IPG). We are then able to map these 15 unique interventions into meaningful categories using the key factors from our proposed framework, as shown in Figure 3.

## Discussion

In this study, we consolidated the wide range of evidence to produce a simple framework to aid the understanding and prevention of caregiver depression in dementia. Our framework underscores the need for prevention efforts to focus on the strategic areas which include the physical-care demands of PWD, behavioral problems of PWD, caregiving competency, and loss and grief of caregivers.

The predictors reported in this study (namely, role as primary caregiver, caring for PWD with later stage of disease, caring for PWD with severe behavioral problems, lower education level, and spousal relationship) are consistent with what has been described in the literature (Schoenmakers *et al.*, 2010; Sallim *et al.*, 2015). This finding gives assurance to the validity of our derived predictors. Notably, our set of predictors bear resemblance to those found to be significant in a recent meta-analysis (Schoenmakers *et al.*, 2010), which included the subjective workload (OR 2.43, 95% CI 2.33–2.53), lower cognitive function and ADL dependence (OR 1.43 and 1.50, respectively, 95% CI 1.24–1.65 and 1.40–1.62, respectively), behavioral disturbances (OR 1.59, 95% CI 1.43–1.77), and spousal relationship (OR 2.25, 95% CI 1.95–2.58). Only two predictors do not overlap between our study and the meta-analysis – the predictor of female caregiver was reported as significant in the meta-analysis (OR 1.62, 95% CI 1.41–1.85) but not replicated in the current study (OR 1.22, 95% CI 0.81–1.83, *p* = 0.344, based on our simple logistic regression), while the predictor of lower education was significant in our study but not established in the meta-analysis.

While our proposed framework in Figure 2 may not be new knowledge to some practitioners in the field, it has a number of strengths which are worth highlighting. First, this framework is iterated from a broad range of evidence related to the predictors, theory, observations, and interventions of caregiver depression. Hence, we can be assured that the framework is sufficiently reflective of the literature at large. Second, the simplicity of this framework means that it can easily be used by busy practitioners and policy-makers to provide evidence-based interventions while relieving them of the need to plow through the extensive literature. Third, despite appearing simple, the framework still reflects the complexities of dementia caregiving and provides a reasonably comprehensive approach to address the various aspects of the caregiver-PWD dyad. By adopting this framework into our routine practice, it ensures that we can be systematic in providing our care and that we do not neglect any crucial elements in our services. This tool may be especially useful to practitioners who do not specialize in dementia care but may sometimes still have PWD under their care, such as the primary care physicians, residents under training, and generalist psychiatrists.

Both the framework in Figure 2 and the list of interventions in Figure 3 can be used in tandem to improve our prevention efforts of caregiver depression. The framework provides the broad structure to inform us of the strategic areas of prevention, while the examples of interventions in Figure 3 can serve to steer the prevention efforts. For instance, to address the physical-care demands and behavioral problems of PWD, we may need to focus on availing the resources for environmental modification, developing programs for PWD to support their cognitive deficits and maintain their well-being, improving community resources related to dementia care, coordinating the care for the caregiver-PWD dyad, and providing clinical guidelines for judicious use of medications in dementia care. Likewise, to improve caregiving competency and address the experience of loss and grief in caregivers, we may need to review the available caregiver programs to incorporate the key interventions as listed in Figure 3.

Some limitations of the study are noteworthy. First, the predictors were derived from the dataset of a cross-sectional study, and hence their causal relationship with caregiver depression may not be demonstrable. Second, the caregivers in this study were recruited from tertiary hospitals and would have possibly received some form of services which were aimed at addressing caregiver depression. It is possible that the predictors derived from these caregivers may be different from those derived from caregivers who have never received specialized services for caregivers. However, this is less likely a concern considering that our derived predictors were not inconsistent with those reported in extant literature (Schoenmakers *et al.*, 2010). Third, we did not directly measure the degree of behavioral problems in PWD. The indirect measure of the need for admission to a geriatric psychiatry ward represented the more severe degree of behavioral problems not manageable in the community setting, which explained the relatively higher OR of behavioral problems in the multiple logistic regression (Table 1). Fourth, our efforts to link the predictors with the underpinning theory required exploration of the literature in search of the relevant mediating factors. While this step involved some subjectivity, we described our iterative process in detail to allow readers to judge whether the process is well-founded. Moreover, the plausibility of the framework is affirmed when it fitted well with known interventions for caregiver depression in the literature. Fifth, the framework we proposed is not prescriptive in nature, and is more useful as a structure to guide the prevention of caregiver depression. This framework will benefit from future intervention studies to assess its validity and efficacy.

## Conflict of interest

None.

## Description of authors’ roles

TML designed the study, planned the statistical analyses, collected the data, performed statistical analyses, interpreted the results, and wrote the paper. JY searched the literature, interpreted the results, and contributed to the manuscript writing. PY advised on the study design, contributed to data collection, interpreted the results, and revised the paper. MG performed statistical analyses, interpreted the results, and reviewed the paper. All authors approved the final version of the paper for submission.

## Supplementary material

For supplementary material accompanying this paper visit http://dx.doi.org/10.1017/S1041610217002629. click here to view supplementary material
