# Supplementary material for: Iterating a framework for the prevention of caregiver depression in dementia: a multi-method approach
Source: Int Psychogeriatr. 2017 Dec 10;30(8):1119–30. doi: 10.1017/S1041610217002629 (PMC6128011; doi:10.1017/S1041610217002629)
Supplement: Supplementary file 1 [file S1041610217002629sup001.docx]

**Supplementary Material**

**Supplementary Appendix A1.** Search strategy for PubMed, Embase and PsycINFO in our umbrella systematic review

**PubMed**

1. dementia[MeSH]

2. "Alzheimer Disease"[MeSH]

3. dementia[title/abstract])

4. 1 or 2 or 3

5. caregiver[MeSH]

6. caregiv*[title/abstract]

7. carer*[title/abstract])

8. 5 or 6 or 7

9. "Adaptation, Psychological"[MeSH]

10. cope[title/abstract]

11. coping[title/abstract]

12. adapt*[title/abstract])

13. 9 or 10 or 11 or 12

14. advance*[title/abstract]

15. severe[title/abstract]

16. late[title/abstract]

17. later[title/abstract]

18. 14 or 15 or 16 or 17

19. stage[title/abstract])

20. 18 and 19

21. behavio*[title/abstract]

22. problem*[title/abstract])

23. 21 and 22

24. primary[title/abstract]

25. education*[title/abstract]

26. spous*[title/abstract])

27. 20 or 23 or 24 or 25 or 26

28. systematic[sb]

29. 4 and 8 and 13 and 27 and 28

**Embase**

1. 'dementia'/exp

2. 'alzheimer disease'/exp

3. dementia:ab,ti

4. alzheimer*:ab,ti

5. 1 or 2 or 3 or 4

6. 'caregiver'/exp

7. caregiv*:ab,ti

8. carer*:ab,ti

9. 6 or 7 or 8

10. 'adaptive behavior'/exp

11. 'coping behavior'/exp

12. cope:ab,ti

13. coping:ab,ti

14. adapt*:ab,ti

15. 12 or 13 or 14

16. 10 or 11 or 15

17. ((advance* OR severe OR late*) NEAR/5 stage):ab,ti

18. (behavio* NEAR/5 problem*):ab,ti

19. primary:ab,ti

20. education*:ab,ti

21. spous*:ab,ti

22. 17 or 18 or 19 or 20 or 21

23. 'systematic review'/exp

24. 5 and 9 and 16 and 22 and 23

**PsycINFO**

1. exp DEMENTIA/

2. exp ALZHEIMER'S DISEASE/

3. dementia.ab,ti

4. Alzheimer*.ab,ti

5. 3 or 4

6. 1 or 2 or 5

7. exp CAREGIVERS/

8. caregiv*.ab,ti

9. carer*.ab,ti

10. 8 or 9

11. 7 or 10

12. exp COPING BEHAVIOR/

13. exp Adaptive Behavior/

14. cope.ab,ti

15. coping.ab,ti

16. adapt*.ab,ti

17. 14 or 15 or 16

18. 12 or 13 or 17

19. ((advance* OR severe OR late*) adj5 stage).ab,ti

20. (behavio* adj5 problem*).ab,ti

21. primary .ab,ti

22. education*.ab,ti

23. spous*.ab,ti

24. 19 or 20 or 21 or 22 or 23

25. exp "Literature Review"/

26. 6 and 11 and 18 and 24 and 25

| **Supplementary Appendix A2** Individual components of interventions for caregiver depression in dementia, using the 33 randomized controlled trials identified by a recent systematic review [[1](#_ENREF_1)] | | | | | | | | | | | | | | | |
| --- | --- | --- | --- | --- | --- | --- | --- | --- | --- | --- | --- | --- | --- | --- | --- |
| Randomized controlled trial (First author and year) | Interventions | | | | | | | | | | | | | | |
|  | Environmental modification | Strategies to compensate for cognitive deficits and maintain the well-being of persons with dementia | Access to community resources | Coordinated care management | Appropriate use of cholinesterase inhibitors and psycho-active drugs | Basic education about dementia | Communication skills with persons with dementia | Behavioral management techniques | Problem solving techniques | Stress management techniques | Cognitive restructuring techniques | Training on nursing care | Future care planning | Support group | Individual counseling to provide emotional support |
| Beauchamp 2005 [[2](#_ENREF_2)] |  |  |  |  |  |  | ● | ● | ● | ● |  |  |  |  |  |
| Belle 2006 [[3](#_ENREF_3)] |  |  | ● |  |  |  |  | ● |  | ● | ● |  |  | ● |  |
| Bruvik 2013 [[4](#_ENREF_4)] |  |  |  |  |  | ● |  |  | ● | ● |  |  |  |  |  |
| Callahan 2006 [[5](#_ENREF_5)] |  | ● |  | ● | ● | ● | ● | ● |  | ● |  |  | ● |  |  |
| Charlesworth 2008 [[6](#_ENREF_6)] |  |  |  |  |  |  |  |  |  |  |  |  |  |  | ● |
| Chu 2011 [[7](#_ENREF_7)] |  |  | ● |  |  |  | ● | ● |  | ● |  |  | ● | ● |  |
| Czaja 2013 [[8](#_ENREF_8)] |  |  | ● |  |  | ● | ● | ● |  | ● |  |  |  | ● |  |
| DeRotrou 2011 [[9](#_ENREF_9)] |  |  | ● |  |  |  | ● | ● | ● | ● |  |  |  |  |  |
| Dias 2008 [[10](#_ENREF_10)] |  |  | ● | ● | ● | ● |  | ● |  | ● |  |  |  | ● | ● |
| Fortinsky 2009 [[11](#_ENREF_11)] |  |  | ● | ● |  | ● |  | ● | ● | ● |  |  | ● | ● |  |
| Gallagher- Thompson 2008 [[12](#_ENREF_12)] |  |  | ● |  |  | ● | ● | ● |  | ● | ● |  | ● |  |  |
| Gallagher Thompson 2010 [[13](#_ENREF_13)] |  |  | ● |  |  | ● |  | ● |  | ● |  |  | ● |  |  |
| Garand 2014 [[14](#_ENREF_14)] |  |  |  |  |  |  |  |  | ● |  |  |  |  |  |  |
| Graff 2007 [[15](#_ENREF_15)] | ● | ● |  |  |  |  |  |  | ● | ● |  |  |  |  |  |
| Jansen 2011 [[16](#_ENREF_16)] |  |  | ● | ● |  |  | ● | ● |  | ● |  | ● |  |  |  |

| Randomized controlled trial (First author and year)  First author and year | Interventions | | | | | | | | | | | | | | |
| --- | --- | --- | --- | --- | --- | --- | --- | --- | --- | --- | --- | --- | --- | --- | --- |
|  | Environmental modification | Strategies to compensate for cognitive deficits and maintain the well-being of persons with dementia | Access to community resources | Coordinated care management | Appropriate use of cholinesterase inhibitors and psycho-active drugs | Basic education about dementia | Communication skills with persons with dementia | Behavioral management techniques | Problem solving techniques | Stress management techniques | Cognitive restructuring techniques | Training on nursing care | Future care planning | Support group | Individual counseling to provide emotional support |
| Joling 2012 [[17](#_ENREF_17)] |  |  | ● |  |  | ● |  | ● |  | ● |  |  |  |  |  |
| Judge 2013 [[18](#_ENREF_18)] | ● | ● | ● |  |  | ● | ● | ● |  | ● | ● |  |  |  |  |
| Kajiyama 2013 [[19](#_ENREF_19)] |  |  | ● |  |  | ● | ● | ● |  | ● | ● |  | ● |  |  |
| Kuo 2013 [[20](#_ENREF_20)] |  |  | ● |  |  |  |  | ● |  |  |  |  |  |  |  |
| Kurz 2010 [[21](#_ENREF_21)] |  |  | ● |  |  | ● |  | ● |  |  |  | ● | ● |  |  |
| Kurz 2012 [[22](#_ENREF_22)] |  | ● |  |  |  |  |  |  |  |  |  |  |  |  |  |
| Lam 2010 [[23](#_ENREF_23)] | ● | ● | ● | ● |  |  | ● | ● |  |  |  |  |  |  |  |
| Livingston 2013 [[24](#_ENREF_24)] |  |  |  |  |  |  | ● | ● |  | ● | ● |  | ● |  |  |
| Logsdon 2011 [[25](#_ENREF_25)] |  |  |  |  |  |  |  |  |  |  |  |  |  | ● |  |
| Losada 2011 [[26](#_ENREF_26)] | ● | ● |  |  |  |  |  |  |  | ● | ● |  |  |  |  |
| Martin Carrasco 2009 [[27](#_ENREF_27)] |  |  |  |  |  | ● |  | ● | ● | ● | ● |  |  |  |  |
| Martin Carrasco 2014 [[28](#_ENREF_28)] |  |  |  |  |  |  | ● | ● | ● | ● | ● |  | ● |  |  |
| Spijker 2013 [[29](#_ENREF_29)] |  |  | ● |  |  |  |  | ● |  |  |  |  |  |  | ● |
| Teri 2005 [[30](#_ENREF_30)] |  |  |  |  |  |  | ● | ● |  | ● |  |  |  |  |  |
| Torkamani 2014 [[31](#_ENREF_31)] |  |  |  |  |  | ● |  |  |  | ● |  |  |  | ● |  |
| Waldorff 2012, Phung 2013 [[32](#_ENREF_32)] |  | ● | ● |  |  | ● |  |  |  | ● |  |  | ● | ● |  |
| Winter 2006 [[33](#_ENREF_33)] |  |  |  |  |  |  |  |  | ● | ● | ● |  |  | ● |  |
| Woods 2012 [[34](#_ENREF_34)] |  | ● |  |  |  |  |  |  |  |  |  |  |  |  |  |

**Additional References:**

1. Weinbrecht A, Rieckmann N, Renneberg B. Acceptance and efficacy of interventions for family caregivers of elderly persons with a mental disorder: a meta-analysis. International psychogeriatrics / IPA. 2016 Jun 8:1-15.

2. Beauchamp N, Irvine AB, Seeley J, Johnson B. Worksite-based internet multimedia program for family caregivers of persons with dementia. The Gerontologist. 2005 Dec;45(6):793-801.

3. Belle SH, Burgio L, Burns R, Coon D, Czaja SJ, Gallagher-Thompson D, et al. Enhancing the quality of life of dementia caregivers from different ethnic or racial groups: a randomized, controlled trial. Annals of internal medicine. 2006 Nov 21;145(10):727-38.

4. Bruvik FK, Allore HG, Ranhoff AH, Engedal K. The effect of psychosocial support intervention on depression in patients with dementia and their family caregivers: an assessor-blinded randomized controlled trial. Dementia and geriatric cognitive disorders extra. 2013;3(1):386-97.

5. Callahan CM, Boustani MA, Unverzagt FW, Austrom MG, Damush TM, Perkins AJ, et al. Effectiveness of collaborative care for older adults with Alzheimer disease in primary care: a randomized controlled trial. Jama. 2006 May 10;295(18):2148-57.

6. Charlesworth G, Shepstone L, Wilson E, Reynolds S, Mugford M, Price D, et al. Befriending carers of people with dementia: randomised controlled trial. Bmj. 2008 Jun 07;336(7656):1295-7.

7. Chu H, Yang CY, Liao YH, Chang LI, Chen CH, Lin CC, et al. The effects of a support group on dementia caregivers' burden and depression. Journal of aging and health. 2011 Mar;23(2):228-41.

8. Czaja SJ, Loewenstein D, Schulz R, Nair SN, Perdomo D. A videophone psychosocial intervention for dementia caregivers. The American journal of geriatric psychiatry : official journal of the American Association for Geriatric Psychiatry. 2013 Nov;21(11):1071-81.

9. de Rotrou J, Cantegreil I, Faucounau V, Wenisch E, Chausson C, Jegou D, et al. Do patients diagnosed with Alzheimer's disease benefit from a psycho-educational programme for family caregivers? A randomised controlled study. International journal of geriatric psychiatry. 2011 Aug;26(8):833-42.

10. Dias A, Dewey ME, D'Souza J, Dhume R, Motghare DD, Shaji KS, et al. The effectiveness of a home care program for supporting caregivers of persons with dementia in developing countries: a randomised controlled trial from Goa, India. PloS one. 2008 Jun 04;3(6):e2333.

11. Fortinsky RH, Kulldorff M, Kleppinger A, Kenyon-Pesce L. Dementia care consultation for family caregivers: collaborative model linking an Alzheimer's association chapter with primary care physicians. Aging & mental health. 2009 Mar;13(2):162-70.

12. Gallagher-Thompson D, Gray HL, Dupart T, Jimenez D, Thompson LW. Effectiveness of Cognitive/Behavioral Small Group Intervention for Reduction of Depression and Stress in Non-Hispanic White and Hispanic/Latino Women Dementia Family Caregivers: Outcomes and Mediators of Change. Journal of rational-emotive and cognitive-behavior therapy : RET. 2008 Dec 01;26(4):286-303.

13. Gallagher-Thompson D, Wang PC, Liu W, Cheung V, Peng R, China D, et al. Effectiveness of a psychoeducational skill training DVD program to reduce stress in Chinese American dementia caregivers: results of a preliminary study. Aging & mental health. 2010 Apr;14(3):263-73.

14. Garand L, Rinaldo DE, Alberth MM, Delany J, Beasock SL, Lopez OL, et al. Effects of problem solving therapy on mental health outcomes in family caregivers of persons with a new diagnosis of mild cognitive impairment or early dementia: a randomized controlled trial. The American journal of geriatric psychiatry : official journal of the American Association for Geriatric Psychiatry. 2014 Aug;22(8):771-81.

15. Graff MJ, Vernooij-Dassen MJ, Thijssen M, Dekker J, Hoefnagels WH, Olderikkert MG. Effects of community occupational therapy on quality of life, mood, and health status in dementia patients and their caregivers: a randomized controlled trial. The journals of gerontology Series A, Biological sciences and medical sciences. 2007 Sep;62(9):1002-9.

16. Jansen AP, van Hout HP, Nijpels G, Rijmen F, Droes RM, Pot AM, et al. Effectiveness of case management among older adults with early symptoms of dementia and their primary informal caregivers: a randomized clinical trial. International journal of nursing studies. 2011 Aug;48(8):933-43.

17. Joling KJ, van Marwijk HW, Smit F, van der Horst HE, Scheltens P, van de Ven PM, et al. Does a family meetings intervention prevent depression and anxiety in family caregivers of dementia patients? A randomized trial. PloS one. 2012;7(1):e30936.

18. Judge KS, Yarry SJ, Looman WJ, Bass DM. Improved Strain and Psychosocial Outcomes for Caregivers of Individuals with Dementia: Findings from Project ANSWERS. The Gerontologist. 2013 Apr;53(2):280-92.

19. Kajiyama B, Thompson LW, Eto-Iwase T, Yamashita M, Di Mario J, Marian Tzuang Y, et al. Exploring the effectiveness of an internet-based program for reducing caregiver distress using the iCare Stress Management e-Training Program. Aging & mental health. 2013;17(5):544-54.

20. Kuo LM, Huang HL, Huang HL, Liang J, Chiu YC, Chen ST, et al. A home-based training program improves Taiwanese family caregivers' quality of life and decreases their risk for depression: a randomized controlled trial. International journal of geriatric psychiatry. 2013 May;28(5):504-13.

21. Kurz A, Wagenpfeil S, Hallauer J, Schneider-Schelte H, Jansen S, Study A. Evaluation of a brief educational program for dementia carers: the AENEAS study. International journal of geriatric psychiatry. 2010 Aug;25(8):861-9.

22. Kurz A, Thone-Otto A, Cramer B, Egert S, Frolich L, Gertz HJ, et al. CORDIAL: cognitive rehabilitation and cognitive-behavioral treatment for early dementia in Alzheimer disease: a multicenter, randomized, controlled trial. Alzheimer disease and associated disorders. 2012 Jul-Sep;26(3):246-53.

23. Lam LC, Lee JS, Chung JC, Lau A, Woo J, Kwok TC. A randomized controlled trial to examine the effectiveness of case management model for community dwelling older persons with mild dementia in Hong Kong. International journal of geriatric psychiatry. 2010 Apr;25(4):395-402.

24. Livingston G, Barber J, Rapaport P, Knapp M, Griffin M, King D, et al. Clinical effectiveness of a manual based coping strategy programme (START, STrAtegies for RelaTives) in promoting the mental health of carers of family members with dementia: pragmatic randomised controlled trial. Bmj. 2013 Oct 25;347:f6276.

25. Logsdon R, Pike K, McCurry S, Hunter P, Maher J, Snyder L, et al. Effectiveness of time-limited support groups for people with early-stage Alzheimer's disease. Alzheimer's & Dementia. 2011;7(4):S279.

26. Losada A, Marquez-Gonzalez M, Romero-Moreno R. Mechanisms of action of a psychological intervention for dementia caregivers: effects of behavioral activation and modification of dysfunctional thoughts. International journal of geriatric psychiatry. 2011 Nov;26(11):1119-27.

27. Martin-Carrasco M, Martin MF, Valero CP, Millan PR, Garcia CI, Montalban SR, et al. Effectiveness of a psychoeducational intervention program in the reduction of caregiver burden in Alzheimer's disease patients' caregivers. International journal of geriatric psychiatry. 2009 May;24(5):489-99.

28. Martin-Carrasco M, Dominguez-Panchon AI, Gonzalez-Fraile E, Munoz-Hermoso P, Ballesteros J, Group E. Effectiveness of a psychoeducational intervention group program in the reduction of the burden experienced by caregivers of patients with dementia: the EDUCA-II randomized trial. Alzheimer disease and associated disorders. 2014 Jan-Mar;28(1):79-87.

29. Spijker A, Teerenstra S, Wollersheim H, Adang E, Verhey F, Vernooij-Dassen M. Influence of adherence to a systematic care program for caregivers of dementia patients. The American journal of geriatric psychiatry : official journal of the American Association for Geriatric Psychiatry. 2013 Jan;21(1):26-36.

30. Teri L, McCurry SM, Logsdon R, Gibbons LE. Training community consultants to help family members improve dementia care: a randomized controlled trial. The Gerontologist. 2005 Dec;45(6):802-11.

31. Torkamani M, McDonald L, Saez Aguayo I, Kanios C, Katsanou MN, Madeley L, et al. A randomized controlled pilot study to evaluate a technology platform for the assisted living of people with dementia and their carers. Journal of Alzheimer's disease : JAD. 2014;41(2):515-23.

32. Waldorff FB, Buss DV, Eckermann A, Rasmussen ML, Keiding N, Rishoj S, et al. Efficacy of psychosocial intervention in patients with mild Alzheimer's disease: the multicentre, rater blinded, randomised Danish Alzheimer Intervention Study (DAISY). Bmj. 2012 Jul 17;345:e4693.

33. Winter L, Gitlin LN. Evaluation of a telephone-based support group intervention for female caregivers of community-dwelling individuals with dementia. American journal of Alzheimer's disease and other dementias. 2006 Dec-2007 Jan;21(6):391-7.

34. Woods RT, Bruce E, Edwards RT, Elvish R, Hoare Z, Hounsome B, et al. REMCARE: reminiscence groups for people with dementia and their family caregivers - effectiveness and cost-effectiveness pragmatic multicentre randomised trial. Health technology assessment. 2012;16(48):v-xv, 1-116.
